# The Validation and Psychometric Properties of the Gaming Instinctual Motivation Scale

**DOI:** 10.3390/ejihpe13090137

**Published:** 2023-09-15

**Authors:** Ai Ni Teoh, Roberto Dillon, Divjyot Kaur

**Affiliations:** 1 School of Social and Health Sciences, James Cook University, Singapore 387380, Singapore; divjyot.kaur@jcu.edu.au; 2 School of Science and Technology, James Cook University, Singapore 387380, Singapore; roberto.dillon@jcu.edu.au

**Keywords:** Gaming Instinctual Motivation Scale, gaming motivation, intention to play games, gaming experience, scale validation

## Abstract

Being able to quantify gaming motivation in a valid, systematic way has important implications for game designers and gaming user experience researchers. In the present study, we aimed to develop and validate a 30-item Gaming Instinctual Motivation Scale (GIMS) based on Dillon’s 6–11 Framework on instinctual gaming motivation and Lazzaro’s gaming experience model. To validate the scale, we recruited 194 regular gamers (*M_age_* = 22.70 years old, *SD* = 4.38) to complete the GIMS based on their general gaming experience and their experience playing role-laying games (RPGs), first-person shooters (FPSs), real-time strategy, puzzle, and action games. We used a cross-validation approach and performed exploratory factor analysis and confirmatory factor analysis to test the structure of the scale and the reliability and validity of the scale, respectively. The final version of the GIMS had a one-dimensional structure with 15 items. It also had good construct validity, χ^2^ (*N* = 117, *df* = 86) = 126.28, *p* = 0.003, CFI = 0.93, TLI = 0.92, and RMSEA = 0.064 (90% CI [0.04, 0.09]), and reliability (CR = 0.89), and an acceptable convergent validity (AVE = 0.35). The one-dimensional structure was generalizable to RPG and FPS games, demonstrating the applicability of the scale to these two gaming genres. Higher scores on the GIMS were also associated with a greater intention to play games.

## 1. Introduction

The gaming industry attracts much revenue from teenagers and young adults every year [1,2,3]. What motivates people in gaming is important for industry professionals to design games that cater to the demands of consumers. To study gaming motivation systematically, a scale assessing gaming motivation is much needed. Since only a limited number of validated scales are available in the literature, the present study aimed to develop and validate a scale that measures gaming motivation.

### 1.1. Gaming Motivation Theories

Thanks to constant evolution and updating of its business models across the past fifty years [4], the gaming industry brought in USD 173 billion in revenue in 2020, of which 57% was contributed by mobile gaming [1]. This is larger than the revenue brought in by global movies and the sports industry in North America combined [3]. The video-game players who contributed to such large revenues were mostly younger than 34 years old [2]; 73% of 6–10-year-olds played video games, along with as many as 84% of 11–14-year-olds and 74% of 15–24-year-olds [2]. With such high stakes linked to both revenue and increasing interest in gaming across individuals of varied ages, it is important to understand the underlying motivations behind gaming. For this, we need reliable measures before in-depth research can be carried out to further develop the lucrative and exciting gaming industry.

What motivates people in gaming is a question of interest to industry professionals, as it can shed light on game design. The Gaming Motivation Scale (GAMS) [5] is a validated questionnaire measuring gaming motivation. However, the questionnaire is based on the self-determination theory [6] and, hence, focuses on general psychological needs, with some adaptations to fit the context of gaming. Questionnaires that measure gaming motivation from the perspective of the gaming experience are rarely found. Therefore, we aimed to develop a gaming motivation questionnaire that stems from the gaming experience.

To develop a scale that stems from an individual’s gaming experience, it is important to understand the theories of gaming motivation. The Presence–Involvement–Flow Framework [7] conceptualizes three components of gaming experience: presence (attention to the gaming world), involvement (the gamer’s motivation), and flow (evaluation of the game). Although motivation is included in this conceptualization, this theory does not provide further elaboration on the motivation(s) behind gaming.

With a focus on the gaming experience, Dillon [8] provided an explicit elaboration on gaming motivation in his 6–11 Framework that describes 6 main emotions that can be induced by gaming and 11 core instinctual motivations for gaming. The 11 instinctual gaming motivations include *survival* (fight-or-flight), *self-identification, collection, greed, protection/care/nurture, aggressiveness, revenge, competition, communication, exploration/curiosity,* and *color appreciation* (see Dillon [8] for details). Although these core instinctual motivations are indeed comprehensive, a measure based on this theory would include 11 subscales that relate to each instinctual motivation. This poses a challenge for scale development, as measures with too many subscales may succumb to issues such as lack of replicability and interpretability [9].

The gaming experience has also been summarized by Lazzaro [10] into four types of fun evoked during gaming. Gamers gain Easy Fun by exploring possibilities and opportunities in the game, while Hard Fun is associated with a sense of achievement gained through overcoming challenges in the game. Serious Fun gives gamers meaning and value in the game, and finally, People Fun is gained through amusement and interacting with other players in the game. These four types of fun can be a useful guide in constructing a gaming motivation scale. However, much insight is needed to understand which aspects of gaming activities give rise to each type of fun. We believe Dillon’s [8] 11 core instinctual motivations are helpful in shedding light in this regard. As such, the 11 core instinctual motivations proposed by Dillon [8] could be considered in conjunction with the four types of gaming fun proposed by Lazzaro [10] for the development of a scale that highlights the overall gaming experience.

### 1.2. The Four Dimensions of Gaming Instinctual Motivation

The 11 instinctual motivations proposed by Dillon [8] can form four dimensions. The first dimension, which we labelled as Heroism, includes instinctual motivations of *survival*, *self-identification*, *protection/care*, *aggressiveness*, and *revenge*. Heroism is an important motivation in gaming, as it may bring meaning and value to gamers and induce the Serious Fun proposed by Lazzaro [10]. Heroism refers to acts of selflessness to sacrifice oneself for the wellbeing of others [11]. It involves fighting challenging situations to *survive*. Dealing with such challenges requires either fighting or escaping from the situation by judging how demanding the situation is and our capacity to deal with the demand [12]. Also, heroes need to demonstrate *aggressiveness* by conquering or killing as a means of defense or attack [8]. Such aggressive acts serve the purpose of *revenge* in response to failures in achieving goals and injustice in virtual social systems [8], as well as *protecting* team members and the needy.

Some games allow players to create their own avatar. Players tend to create an avatar with an image closer to their ideal self, rather than their actual self, and will gradually *identify* with the avatar and feel connected to the avatar [13]. Trans and gender-diverse players may also benefit mentally from customizing avatars in video games, as this can facilitate their gender identity expression [14]. Challenges created in video games provide opportunities for heroic acts that inspire players to see their avatars in video games as heroes [15]. Such an image attached to the avatar brings important implications to players’ self-image in real life [16].

The second category (Collection) comprises the instinctual motivations of *collection* and *greed*, which can create the Easy Fun proposed by Lazzaro [10]. The instinctual motivation of *collection* originates from the human hunting instinct [17] to acquire and keep prey to symbolize one’s capability and power [18,19]. Although possessing items shapes our identity [20], we believe that *self-identification* would be more relevant to Heroism than Collection because virtual items collected in games might not be as impactful as physical items. *Greed* is another reason why we are motivated to collect. In satisfying our psychological dissatisfaction, we become greedy for resources and motivated to acquire and keep as much as we possibly can [21]. Video games usually offer performance-based rewards or treasures to motivate players. Players are therefore motivated to explore and maximize benefits from opportunities [22]. Therefore, they aspire to perform well so as to receive those treasures that are helpful in upgrading to the next level.

The third category (Quest) comprises the instinctual motivations of *competition* and *curiosity*, which can predict the Hard Fun described by Lazzaro [10]. Video games create challenges for gamers to overcome. Oftentimes, to overcome the challenges, players need to *compete* individually or collectively with their team members. Having a sense of *curiosity* may increase competitiveness. Curiosity is induced intrinsically [23] in situations that violate expectations. Driven by curiosity, players will try to make sense of situations and gain control over them. Gaining an understanding and control of challenging situations helps players overcome the situations and gain a sense of achievement (Hard Fun).

The fourth category (Personal Experience) comprises the instinctual motivations of *communication* and *color appreciation*, which can induce the People Fun described by Lazzaro [10]. Game players might need to *communicate* with other human players or virtual characters, which generally involves asking for and providing information and suggestions [24]. Over time, such communication may help players form friendships with other human players or virtual players [24,25]. The friendship with human players may extend to offline friendships and frequent face-to-face social gatherings [25,26]. In addition to social interactions, gamers are motivated by *color appreciation* to play games. The vividness and novelty of scenes and artistic color use are important elements that attract gamers [16,27].

### 1.3. Research Aims

There are few questionnaires that measure gaming motivation from the perspective of gaming experience. As such, researchers and game developers might be restricted by the limited number of validated scales available to systematically study gaming motivation. In view of both, the need for a validated scale is critical for research in this area. With this purpose in mind, in an initial study [28], we developed a 30-item Gaming Instinctual Motivation Scale (GIMS) and tested its face validity and internal consistency using a small sample of 20 participants.

In the present study, we aimed to further test the validity and structure of the scale using a larger sample by conducting exploratory factor analysis (EFA) and confirmatory factor analysis (CFA).

## 2. Materials and Methods

### 2.1. Participants and Design

We planned to recruit about 200 participants. This sample size would prove sufficient based on the criteria recommended by Comrey and Lee [29] (*N* of 200 to provide a fair adequacy of sample size) and Gorsuch [30] (five subjects for each item, with a minimum *N* of 100). To recruit participants, we sent out a mass email to all students at the university and posted advertisements on social media platforms. We invited adults who are regular video-game players who play online, mobile, console, and/or computer games to participate in the study. Altogether we recruited 194 participants aged between 18 and 40 years (*M_age_* = 22.70 years old, *SD* = 4.38), with 89 women, 72 men, and 33 who did not specify their gender. The participants spent between 0.5 and 80 h playing video games per week (*M* = 17.60, *SD* = 13.45).

### 2.2. Measures

#### 2.2.1. Gaming Instinctual Motivation Scale (GIMS)

This is a self-constructed questionnaire measuring the 11 instinctual motivations for gaming based on the 6–11 Framework [8] and four types of gaming fun [10]. Participants rated all of the 30 items on a Likert scale from 1 (*never*) to 5 (*always*). See Table 1 for all of the items of the original GIMS. In an initial study [28], we developed the GIMS and showed that the scale had face validity and high internal consistency (between 0.77 and 0.96) across the 17 game genres tested, suggesting that the scale can be used for various game genres.

Participants had to complete this scale six times in the study. When completing the scale for the first time, the participants had to indicate how applicable each item was to them when playing a game *in general*. From the second to sixth times, the participants had to complete the scale based on their experience playing role-playing games (RPGs), first-person shooters (FPSs), real-time strategy (RTS), puzzle, and action games, respectively (with the game genres in a randomized order).

There were 194 participants who completed the GIMS based on their general gaming experience; 76 participants completed the scale based on their experience playing RPGs, 85 participants for FPSs, 26 participants for RTSs, 42 participants for puzzles, and 34 participants for action games. For the purposes of the study, we performed EFA and CFA using the responses that were based on the general gaming experience. We also tested the final model on the data pertaining to RPG and FPS games (given the higher sample sizes) to show the generalizability of the findings to other game genres.

#### 2.2.2. Gaming Motivation Scale (GAMS)

This scale was constructed and validated by Lafreniere and colleagues [5]. It comprises 6 subscales (e.g., intrinsic motivation, amotivation) with 18 items that assess the motivation for playing games. The motivation measured in this scale is based on the self-determination theory [6]. The participants completed the items based on their general gaming experience on a Likert scale of 1 (*do not agree at all*) to 7 (*very strongly agree*). The inclusion of this scale was to test the convergent validity of the GIMS. The scale and subscales of the GAMS had high internal consistency values and construct validity. In the present study, the GAMS had a Cronbach’s alpha value of 0.83.

#### 2.2.3. Gaming Retention Scale

This is a self-conatructed one-item scale that requires participants to rate their intention to continue playing a particular game genre. For each game genre, participants rated on the item “How likely are you going to continue with ______ game?” on a Likert scale from 1 (*not likely*) to 7 (*very likely*).

#### 2.2.4. Demographic Scale

This scale required participants to indicate their gender, age, and the average number of hours that they spent playing video games per week.

In addition to the scales described above, we administered the Positive and Negative Affect Schedule—Extended Form (PANAS-X) [31] in this study. However, it was not within the scope of the present manuscript.

### 2.3. Procedure

Upon ethical approval from James Cook University’s Human Research Ethics Committee (approval number: H7941), we recruited participants via emails and advertisements on social media from November 2019 to June 2020. Students who were interested in participating clicked the link provided in the emails and advertisements. The link directed participants to the Qualtrics online system, which first showed an information sheet about the study, followed by the informed consent. Informed consent was considered to have been given once the participants had read the information sheet and informed consent form and selected the “agree” button to indicate consent. Once they hit the “agree” and “next” buttons, they completed a series of questionnaires. If they hit the “disagree” button, Qualtrics directed them out of the survey.

Participants completed the GIMS and the GAMS in terms of their general gaming experience. After this, an item asked participants to indicate whether they played a particular genre of games (e.g., puzzle games) regularly. If they indicated “yes”, they completed the GIMS, PANAS-X, and Gaming Retention Scale (in a randomized order) based on their experience playing the genre. Next, an item asked participants to indicate whether they played another genre of games (e.g., action/adventure games). Likewise, if they indicated “yes”, they completed the three scales in a randomized order based on their experience playing the genre. The cycle continued for all five genres. Participants skipped to the next genre if they indicated “no” to a game genre. Lastly, they completed the demographic scale. The online survey took about 8 to 20 min to complete, depending on the number of game genres a participant usually played.

### 2.4. Data Analysis Strategies

We used a cross-validation approach to validate the scale. From the original sample size of 194 participants, we randomly split the data into two subsamples. The first subsample (Subsample 1) comprised 77 participants (40% of the sample), which would be used for EFA. A sample size of lower than 50 is sufficient for EFAs with high factor loadings, low numbers of factors, and high numbers of items [32]. The second subsample (Subsample 2) comprised 117 participants (60% of the sample), which would be used for CFA. The 40–60 split was to allow a larger sample size for CFA while providing sufficient sample size for EFA [32].

Using Subsample 1, we performed EFA using the principal axis factoring approach for data extraction with IBM SPSS 27. We extracted factors with eigenvalues larger than 1 and rotated the factors using the direct oblimin method. From the EFA output, we eliminated (1) items that had absolute values of factor loadings lower than 0.40 (a rule of thumb of 0.32, so we decided to set the cutoff at 0.40) [33], and (2) cross-loading (or complex) items that had high factor loadings with two or more factors (i.e., difference in absolute factor loadings lower than 0.20).

With the structure obtained from EFA, we performed CFA using Subsample 2 with IBM SPSS AMOS 27. We modified the model based on the goodness-of-fit indices, including chi-square statistics (χ^2^/*df*; preferably less than 2), comparative fit index (CFI; recommended to be larger than 0.95; can be larger than 0.95 for continuous data), Tucker–Lewis index (TLI; recommended to be larger than 0.95; can be between 0 and 1 for continuous data), and the root-mean-square error of approximation (RMSEA; recommended to be less than 0.05 or 0.08 and significant) [34]. When the indices showed a poor fit, we first removed items with (absolute values of) factor loadings lower than 0.50 and then correlated the residuals based on the modification index. We deemed each step of the modification to be valid if ∆CFI was larger than 0.01.

We computed composite reliability (CR) for the reliability of the factors, average variance extracted (AVE) for convergent validity, and maximum shared squared variance (MSV) for shared variance. CR shows the internal consistency of scale items; AVE estimates the amount of variability in the observed variables that the latent variable can explain; MSV is the square of the highest inter-factor correlations, referring to the variance in observed variables that the other latent variables can explain. To assess the discriminant validity, we compared AVE with MSV and the square root of AVE with inter-factor correlations. The factors with AVE larger than MSV [35] and with a square root of AVE larger than the inter-factor correlations were considered to have high discriminant validity [36].

Given the higher numbers of participants completing the GIMS in the contexts of RPG and FPS games, we conducted SEM to examine the goodness of fit of the final model on each genre. Such findings would shed light on the generalizability of the model to other genres of games.

## 3. Results

### 3.1. Exploratory Factor Analysis

We performed EFA using Subsample 1 to test the structure of the scale. Before performing EFA, we tested the assumptions of EFA. Subsample 1 did not contain any outliers, but the variables were not normally distributed. However, factor analysis is robust against any violation of the normality assumption [33]; therefore, no data transformation was performed. Using eigenvalues larger than 1, the analysis extracted seven factors, but the factor rotation failed to converge. Therefore, we performed another EFA by fixing the number of factors to four. The analysis met the factorability assumption, with the Kaiser–Meyer–Olkin (KMO) measure of sample adequacy larger than 0.60 (KMO = 0.79), Bartlett’s test of sphericity significant, χ^2^ (435) = 1286.78, *p* < 0.001, and anti-image correlations larger than 0.60.

The EFA analysis generated a pattern matrix with four factors (see Table 2). Factor 1 (Heroism) consisted of 11 items. However, we removed four items that were complex items. Factor 2 (Quest) had six items, but we removed three complex items. Factor 3 (Collection) had eight items, three of which were complex items and were removed. Factor 4 (Personal Experience) had five items, only one of which was a complex item and was removed. The four-factor structure was later examined using CFA.

### 3.2. Confirmatory Factor Analysis

After confirming the structure of the scale using EFA, we then performed CFA using Subsample 2 to cross-validate the scale. The initial four-factor structure yielded unsatisfactory model-fit indices, χ^2^ (*N* = 117, *df* = 146) = 265.84, *p* < 0.001, CFI = 0.84, TLI = 0.81, and RMSEA = 0.084 (90% CI [0.07, 0.10]). We then went through three steps to remove items that had factor loadings lower than 0.50, which included “*Punish someone for something*,” “*Nurturing a puppy*,” “*Increasing skills/abilities*,” “*Be a thief or a killer*,” and “*Escorting a friend/companion through a dangerous area*,” and correlated the error terms of “*Conquering the world*” and “*Conquering a territory/area*.” Each step produced ∆CFIs larger than 0.01. The final structure showed a good fit, χ^2^ (*N* = 117, *df* = 83) = 123.09, *p* = 0.003, CFI = 0.93, TLI = 0.91, and RMSEA = 0.065 (90% CI [0.04, 0.09]). In sum, the model was reduced to 15 items.

The four-factor model had satisfactory internal consistency, with Cronbach’s alpha values higher than 0.70. It also had satisfactory reliability, with CR values higher than 0.70 for all four factors (see Table 3). With the goodness-of-fit indices meeting the criteria, the model had high construct validity. The model did not have sufficient convergent validity, with the AVEs for all factors (except for Quest) not exceeding the 0.50 cutoff point. However, construct validity can still be inferred when CR values are larger than 0.60 but AVE is lower than 0.50 [35]. The discriminant validity was not sufficient for the model, since the MSV values were larger than the AVEs, and the square roots of the AVEs were less than the inter-factor correlations. The model did not meet the criteria, showing insufficient discriminant validity. In other words, a factor (e.g., Heroism) explained more of the variance in the items of other factors (e.g., Collection) than the variance of their own items.

To deal with the lack of discriminant validity, it is advisable to check the EFA results again to identify and remove cross-loading items or to combine the latent variables into one overall measure [36]. Since we attempted the first option before performing the CFA by removing cross-loading items, at this stage we chose the second option and examined the one-factor solution.

**Figure 1:**
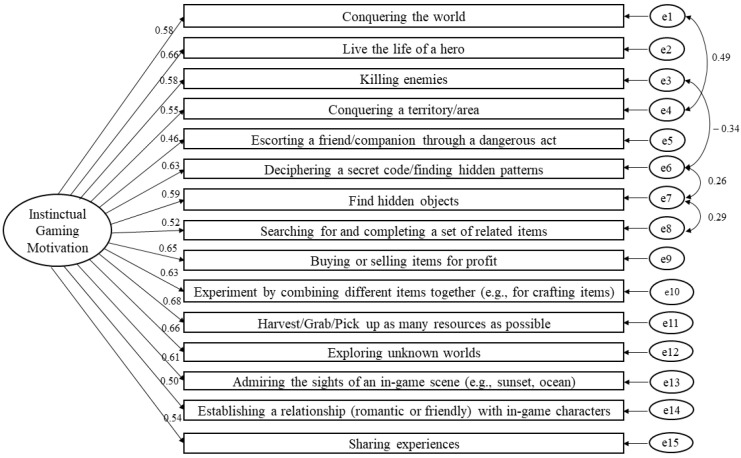
The one-dimensional model of the Gaming Instinctual Motivation Scale using confirmatory factor analysis based on the general gaming experience (standardized estimates).

The initial model showed a poor fit, χ^2^ (*N* = 117, *df* = 89) = 158.77, *p* < 0.001, CFI = 0.88, TLI = 0.86, and RMSEA = 0.082 (90% CI [0.06, 0.10]). After correlating three pairs of error terms, the model showed a good fit, χ^2^ (*N* = 117, *df* = 86) = 126.28, *p* = 0.003, CFI = 0.93, TLI = 0.92, and RMSEA = 0.064 (90% CI [0.04, 0.09]). The goodness-of-fit indices showed that the one-factor model had good construct validity. It also had good internal consistency (α = 0.89) and reliability (CR = 0.89). The convergent validity was not satisfactory (AVE = 0.35) but was acceptable given the high CR [35]. See Figure 1 for the structure of the model.

We averaged the GIMS scores for each participant based on the final model and compared the average scores with the scores of the subscales of the GAMS. The GIMS was positively associated with the intrinsic motivation scores of the GAMS, showing that the GIMS focuses mainly on intrinsic motivation. The correlation showed an acceptable convergent validity (see Table 4). The GIMS was also positively associated with the intention to continue playing the RPG, FPS, RTS, and action games that they identified (see Table 5).

### 3.3. Generalizability to Other Genres of Games

**Figure 2:**
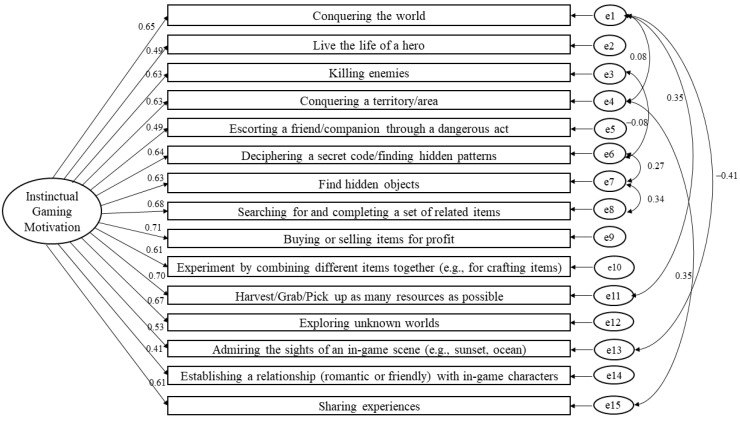
The one-dimensional model of the Gaming Instinctual Motivation Scale using confirmatory factor analysis based on the experience playing RPGs (standardized estimates).

We examined the applicability of the 15-item GIMS to RPGs. There were 76 participants who completed the GIMS based on their experience playing RPGs. This sample size is sufficient given the five-subjects-per-item criterion recommended by Gorsuch [30]. The results showed that the scale, when applied to RPGs, had high construct validity (see Figure 2). Although it had a poor fit in the initial model, χ^2^ (*N* = 76, *df* = 86) = 151.59, *p* < 0.001, CFI = 0.85, TLI = 0.82, and RMSEA = 0.101 (90% CI [0.07, 0.13]), correlating additional three pairs of errors enhanced the goodness of fit, χ^2^ (*N* = 76, *df* = 83) = 119.19, *p* = 0.006, CFI = 0.92, TLI = 0.90, and RMSEA = 0.076 (90% CI [0.04, 0.11]). The model also had good reliability (CR = 0.89) and internal consistency (α = 0.90). The low convergent validity (AVE = 0.38) was not an issue given the high CR [35].

**Figure 3:**
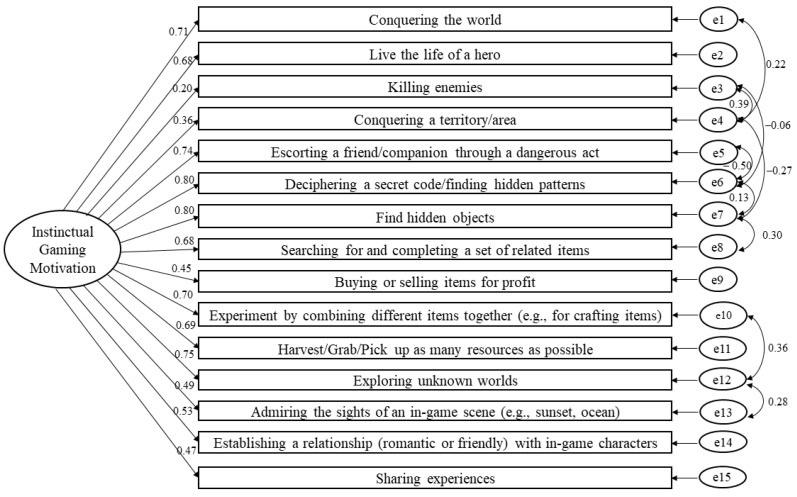
The one-dimensional model of the Gaming Instinctual Motivation Scale using confirmatory factor analysis based on the experience playing FPS games (standardized estimates).

There were 85 participants who completed the GIMS based on their experience playing FPS games, a sample size sufficient for a CFA [30]. Therefore, we conducted a CFA on this dataset. When applied to FPS games, the 15-item GIMS had high construct validity as well (see Figure 3). The initial model, which had a poor fit, χ^2^ (*N* = 85, *df* = 86) = 180.39, *p* < 0.001, CFI = 0.83, TLI = 0.79, and RMSEA = 0.114 (90% CI [0.09, 0.14]), had its goodness-of-fit indices greatly improved after correlating five pairs of errors, χ^2^ (*N* = 85, *df* = 81) = 121.40, *p* = 0.002, CFI = 0.93, TLI = 0.91, and RMSEA = 0.077 (90% CI [0.05, 0.10]). The internal consistency was satisfactory (α = 0.91). The reliability was high (CR = 0.90) and, hence, could compensate for the low convergent validity (AVE = 0.39) [35].

## 4. Discussion

### 4.1. Key Findings

The final version of the GIMS was reduced to 15 items. The data showed a good fit with the one-dimensional structure. We found good construct validity and acceptable convergent validity for the scale. The scale also had acceptable convergent validity with the intrinsic motivation subscale of the GAMS [5]. The one-dimensional structure also showed generalizability to the two gaming genres that we tested, demonstrating the applicability of the scale to RPG and FPS games, if not all gaming genres. Higher GIMS scores were associated with a greater intention to continue playing RPG, FPS, RTS, and action games.

### 4.2. Theoretical Implications

We constructed the GIMS by referring to Lazzaro’s [10] four types of gaming fun and Dillon’s [8] 11 core instinctual motivations. Our items were constructed based on the 11 instinctual motivations, and we expected that the 11 motivations could be categorized into four factors, which are the four types of gaming fun. Contrary to our expectation, the scale had insufficient discriminant validity for the four-factor structure. A one-factor structure, on the other hand, showed a good fit and good validity and reliability. The lack of discriminant validity in the four-factor structure suggested that the latent variables were highly correlated [37]. Such a finding seems reasonable, as there appears to be much overlap among the 11 core instinctual motivations [8].

For instance, Heroism was highly associated with Quest and Collection in the present study (see Table 3). Consistent with the literature, Heroism, Quest, and Collection could be connected via self-identity. Players identify themselves with their virtual identity or the avatar selected, especially when the identity is idealized [38]. Identification may also occur when players associate avatars’ heroic acts and quests with themselves and identify themselves with the heroic avatar [15]. In addition, players may form self-identity in video games by possessing treasures, fulfilling their motivation of self-enhancement [18].

Also, Heroism, Quest, Collection, and Personal Experience were interrelated in the present study (see Table 3), which is consistent with the literature. For instance, to accomplish Heroism, players need to increase their Collection, widening their pool of resources to strengthen their combat power. With more resources, they have a greater capacity to avenge and protect, among other heroic acts. To increase Collection, players may need to compete with other groups in completing Quests and cooperate with team members (Personal Experience) to complete Quests. Competing with other groups in Quests, in turn, requires greediness [39] or collecting more treasures and resources than needed in the context of video gaming (Collection). Quest completion can also be facilitated by increasing curiosity in players so as to resolve uncertainty and gain control [23]. To increase curiosity, video games increase players’ motivation to collect (Collection) and form self-identification (Heroism) [8]. Overall, it appears that Heroism, Quest, Collection, and Personal Experience are intertwined. Therefore, the high correlations among the factors and, hence, the insufficient discriminant validity in the findings are not completely unexpected.

### 4.3. Practical Implications

Cyberpsychology is a relatively young research area, so researchers often have to self-develop scales to measure gaming-related psychological constructs—an approach that compromises the validity and reliability of research findings [40]. The GIMS fills the gap in the literature where limited scales on gaming motivation are available. The scale takes on the perspective of gaming experience and motivation, making it different from other existing scales. It has 15 items without any subscales. Higher scores represent a higher level of instinctual gaming motivation.

The GIMS could be useful in informing professional game designers of players’ motivation(s) to play a game. The items in the GIMS focus on various aspects of the content of games. The scale is composed of the four original factors (Heroism, Quest, Collection, and Personal Experience). Although our findings showed that the four-dimensional structure was not established, the relevant items of each factor may shed some light on how the factor contributes to the overall gaming motivation. A lower score on Quest, for instance, suggests that improving Quest-related activities may be helpful in increasing gaming motivation, which may be desirable if the game being designed is, for example, an RPG.

With gaming motivation being quantified using the GIMS, marketing research related to potential customers’ preferences for games and gaming genres could be facilitated. Researchers can examine how demographic variables affect gaming motivation and how gaming motivation is associated with other variables of interest, such as intention to purchase the game and players’ experiences.

### 4.4. Limitations and Suggestions for Future Studies

The development of the GIMS was theory-based, and the validation of the scale demonstrated good reliability and validity. However, the following limitations of the study might limit the generalizability and interpretation of the findings:

Our cross-validation approach, which split the sample into two subsamples for EFA and CFA, provided a rather low sample size for CFA. The CFAs conducted on the data related to the RPG and FPS genres had low sample sizes as well. Although low, the sample sizes were sufficient for CFA [30]. However, a larger sample size, such as 200, could provide a larger statistical power [29].

Our findings have low generalizability, which might affect how we interpret the findings. Although we tested the generalizability of the scale to the RPG and FPS genres in the present study, many other game genres were not included in the study. Also, we recruited regular gamers whose average hours spent on gaming ranged widely. Therefore, the findings were not generalizable to other types of players, such as players of varied gaming skill levels and professional esports players. Therefore, future studies should test the structure, validity, and reliability of the GIMS in various genres of games using different populations, such as amateur game players, novice game players, and game addicts.

We found a significant, positive association between the GIMS scores and the intention to play games. Future studies could explore the predictability of the GIMS for other outcomes, such as emotions and brain activity. Since certain parts of the brain (e.g., the pathway linking the lateral hypothalamus to the ventral tegmental area) [41] are associated with motivation, higher scores on the GIMS should predict higher activity in those parts of the brain.

## 5. Conclusions

The final version of the GIMS has 15 items grouped into one factor with good reliability and validity. The GIMS was also positively associated with the intention to play games. Future studies may extend from the present study and examine how the GIMS can predict other outcomes, such as the brain regions associated with motivation.

## Figures and Tables

**Table 1:** The 11 instinctual motivations outlined in the 6–11 Framework and the items of the original Gaming Instinctual Motivation Scale.

Instinctual Motivations	Items of the Original Gaming Instinctual Motivation Scale
Survival	Fight unspeakable horrors
	Escape deadly dangers
Self-identification	Live the life of a hero
	Catch a thief/killer
	Complete a journey
	Increasing skills/abilities
Collecting	Searching for and completing a set of related items
	Find hidden objects
	Buying or selling items
Greed	Acquiring power/riches
	Harvest/grab/pick up as many resources as possible
Protection/care	Rescue person in danger
	Saving the world
	Escorting a friend/companion through a dangerous area
	Nurturing a puppy
Aggressiveness	Killing enemies
	Be a thief or a killer
	Conquering a territory
Revenge	Punish someone for something, i.e., take revenge
Competition	Conquering the world
	Defeating another player in a direct confrontation
Communication	Establishing a relationship (romantic or friendly) with in-game characters
	Sharing experiences
	Retrieving information by listening/talking to other characters
Curiosity	Unfolding a mystery
	Experiment by combining different items together (e.g., for crafting items)
	Exploring unknown worlds
	Deciphering a secret code
Color appreciation	Admiring the sights of an in-game scene (e.g., sunset, ocean)
	Fascination/engagement with color and design of in-game items

**Table 2:** The pattern matrix from the exploratory factor analysis.

Items	Factor			
	Heroism	Quest	Collection	Personal Experience
Conquering the world	**0.69 ^a^**	0.03	−0.09	−0.01
Punish someone for something, i.e., take revenge	**0.67 ^a^**	−0.24	−0.07	−0.01
Live the life of a hero	**0.65 ^a^**	0.13	0.10	0.21
Killing enemies	**0.61 ^a^**	−0.18	−0.25	0.03
Conquering a territory/area	**0.53 ^a^**	0.20	−0.20	−0.12
*Acquiring power/riches*	*0.50*	*−0.19*	*−0.49*	*0.12*
Be a thief or a killer	**0.47 ^a^**	0.02	−0.08	0.14
*Saving the world*	*0.46*	*0.31*	*0.01*	*0.21*
*Escape deadly dangers*	*0.45*	*0.33*	*−0.22*	*0.06*
Escorting a friend/companion through a dangerous area	**0.40 ^a^**	0.08	0.03	0.16
*Fight unspeakable horrors*	*0.37*	*0.24*	*−0.22*	*−0.05*
Deciphering a secret code/finding hidden patterns	−0.13	**0.74 ^b^**	−0.18	−0.03
Find hidden objects	0.03	**0.59 ^b^**	−0.29	0.02
Nurturing a puppy	0.06	**0.57 ^b^**	0.14	0.08
*Catch a thief/killer*	*0.41*	*0.55*	*−0.02*	*−0.02*
*Rescue person in danger*	*0.43*	*0.46*	*0.05*	*0.17*
*Unfolding a mystery*	*0.03*	*0.46*	*0.00*	*0.40*
Searching for and completing a set of related items	0.10	0.35	**−0.69 ^c^**	−0.11
Buying or selling items for profit	0.01	−0.14	**−0.59 ^c^**	0.14
Experiment by combining different items together (e.g., for crafting items)	0.04	0.14	**−0.58 ^c^**	0.16
Increasing skills/abilities	0.10	0.08	**−0.50 ^c^**	0.03
Harvest/grab/pick up as many resources as possible	0.12	0.07	**−0.48 ^c^**	0.18
*Defeating another player in a direct confrontation*	*0.34*	*−0.14*	*−0.44*	*−0.16*
*Complete a journey*	*−0.23*	*0.31*	*−0.37*	*0.33*
*Retrieving information by listening/talking to other characters*	*0.25*	*0.01*	*−0.31*	*0.24*
Exploring unknown worlds	−0.15	0.15	−0.29	**0.66 ^d^**
Admiring the sights of an in-game scene (e.g., sunset, ocean)	−0.03	0.08	−0.03	**0.61 ^d^**
Establishing a relationship (romantic or friendly) with in-game characters	0.13	−0.16	0.02	**0.55 ^d^**
Sharing experiences	0.27	−0.01	0.00	**0.50 ^d^**
*Fascination/engagement with color and design of in-game items*	*0.05*	*0.15*	*−0.23*	*0.41*

Note: *N* = 77. Rotation converged in 14 iterations. Items in bold have factor loadings ≥ 0.30. Items in italics are complex items that were removed for confirmatory factor analysis. Items with the same superscript are grouped under the same factor.

**Table 3:** The reliability and validity indicators for confirmatory factor analysis using the four-factor model.

Factor	Cronbach’s Alpha	CR	AVE	MSV	Factor Correlations/Square Roots of AVEs			
					Heroism	Quest	Collection	PE
Heroism	0.79	0.77	0.41	0.73	**0.64**			
Quest	0.72	0.73	0.57	0.76	0.85	**0.76**		
Collection	0.72	0.72	0.39	0.86	0.60	0.87	**0.63**	
PE	0.70	0.71	0.38	0.86	0.73	0.93	0.78	**0.62**
Criterion	>0.60	>0.70	>0.50					

Note: CR = composite reliability; AVE = average variance extracted; MSV = maximum shared variance; PE = Personal Experience. The values in bold are the square roots of the AVEs.

**Table 4:** The correlation coefficients between the GIMS and GAMS subscales.

Scales	GIMS	(GAMS) Intrinsic Motivation	(GAMS) Integrated Regulation	(GAMS) Identified Regulation	(GAMS) Introjected Regulation	(GAMS) External Regulation	(GAMS) Amotivation
GIMS	1.00	−0.17 *	0.09	0.08	0.06	0.05	−0.06
Means	3.38	3.63	3.20	3.34	2.53	3.29	2.82
Standard deviation	0.80	1.30	1.35	1.27	1.14	1.32	1.35

Note: GIMS = Gaming Intrinsic Motivation Scale; GAMS = Gaming Motivation Scale; * *p* < 0.05.

**Table 5:** Correlation coefficients between GIMS scores and intention to continue playing each genre of games.

Intention to Continue		GIMS Scores	Mean	Standard Deviation
How likely are you to continue with first-person shooter games?	*N* = 85	0.35 **	5.78	1.34
How likely are you to continue with role-playing games?	*N* = 76	0.33 **	6.03	1.34
How likely are you to continue with real-time strategy games?	*N* = 26	0.46 *	5.38	1.60
How likely are you to continue with puzzle games?	*N* = 42	0.21	5.55	1.42
How likely are you to continue with action/adventure games?	*N* = 34	0.44 **	6.18	1.06

Note: GIMS = Gaming Intrinsic Motivation Scale; * *p* < 0.05, ** *p* < 0.01.

## Data Availability

The data are available from the corresponding author upon reasonable request.
